# Forecasting the 2021 local burden of population alcohol‐related harms using Bayesian structural time–series

**DOI:** 10.1111/add.14568

**Published:** 2019-03-06

**Authors:** Cheryl McQuire, Kate Tilling, Matthew Hickman, Frank de Vocht

**Affiliations:** ^1^ Population Health Sciences, Bristol Medical School University of Bristol Bristol UK; ^2^ MRC Integrative Epidemiology Unit University of Bristol Bristol UK

**Keywords:** Alcohol, Bayesian statistics, forecasting, hospital admissions, nowcasting, public health, time–series

## Abstract

**Background and aims:**

Harmful alcohol use places a significant burden on health services. Sophisticated nowcasting and forecasting methods could support service planning, but their use in public health has been limited. We aimed to use a novel analysis framework, combined with routine public health data, to improve now‐ and forecasting of alcohol‐related harms.

**Design:**

We used Bayesian structural time–series models to forecast alcohol‐related hospital admissions for 2020/21 (from 2015 to 2016).

**Setting:**

England.

**Participants:**

We developed separate models for each English lower‐tier local authority.

**Measurements:**

Our primary outcome was alcohol‐related hospital admissions. Model covariates were population size and age‐structure.

**Findings:**

Nowcasting validation indicated adequate accuracy, with 5‐year nowcasts underestimating admissions by 2.2% nationally and 3.3% locally, on average. Forecasts indicated a 3.3% increase in national admissions in 2020/21, corresponding to a 0.2% reduction in the crude rate of new admissions, due to population size changes. Locally, the largest increases were forecast in urban, industrial and coastal areas and the largest decreases in university towns and ethnically diverse areas.

**Conclusions:**

In 2020/21, alcohol‐related hospital admissions are expected to increase in urban and coastal areas and decrease in areas associated with inward migration of younger people, including university towns and areas with greater ethnic diversity. Bayesian structural time–series models enable investigation of the future impacts of alcohol‐related harms in population subgroups and could improve service planning and the evaluation of natural experiments on the impact of interventions to reduce the societal impacts of alcohol.

## Introduction

Alcohol is a major cause of morbidity and mortality 1, 2 and heavy alcohol use is particularly common in the United Kingdom. In 2017, 29% of men and 26% of women in Great Britain reported binge drinking and 10% of adults reported drinking alcohol on 5 or more days per week 3. More than 1.1 million hospital admissions (7% of all admissions) and 24 208 deaths in England were attributable to alcohol use 4, 5. This places a significant burden on health‐care services, costing the National Health Service (NHS) an estimated £3.5 billion per year 6. Patterns of alcohol consumption and related harm are complex, however, and vary widely between different time‐periods, geographical regions and population subgroups.

Public health surveillance is crucial for monitoring and understanding the epidemiology of health‐related outcomes, for informing policy and resource allocation and for evaluating the impact of interventions 7. However, there is often a delay between the real‐time occurrence of a health‐related event and the availability of data about that event. Nowcasting, defined as the prediction of the recent past, the present and the near future, has been used to correct for such lags in data availability. Forecasting, in contrast, is important for estimating trends in the longer term 8.

Now‐ and forecasting methods have been used to model trends in disease, including outbreaks of gastrointestinal disease and influenza epidemics, and can support timely responses to public health events 9, 10. Traditional methods, including static regression and autoregressive integrated moving‐average (ARIMA) models have been used widely for now‐ and forecasting, but have important limitations. Under a static regression approach, the time–series is modelled in a deterministic way as the sum of trend, seasonal and irregular components and it assumes that the behaviour of the time–series is stable over time, which is rarely tenable. ARIMA models rely only on the past behaviour of the time–series and previous error terms for forecasting 11. Structural time–series models have been proposed as an alternative approach, as they allow model parameters to evolve with time, can incorporate a wide range of explanatory variables and may more accurately reflect the stochastic nature of the time–series 12. Sophisticated now‐ and forecasting methods are established in areas including meteorology, marketing and economics. Some of these benefit from robust and automatic selection of covariates and incorporate changes in predictor–outcome relationships over time 13. The application of these methodologies within public health could have important implications for prioritization, development and implementation of policies to address and prevent adverse health outcomes.

In this context, we describe a novel analysis framework, Bayesian structural time–series 13, combined with routine public health data, to improve now‐ and forecasting of alcohol‐related harms. We have previously used this methodology to estimate the impact of local alcohol licensing policies on health and crime outcomes within a natural experiment study design, using a causal inference framework 14. In this study, we extend this methodology independently of the causal inference framework to forecast trends in alcohol‐related hospital admissions for different geographic aggregations in England, based on projected local trends in population size and age structure.

## Methods

### Nowcasting the present (model development and validation)

We developed separate Bayesian structural time–series models for each lower‐tier local authority (LTLA) in England and evaluated the accuracy and precision with which each model captured the measured alcohol‐related hospital admissions for 1–5‐year nowcasting periods.

### Forecasting the future

After evaluating nowcasting accuracy, we used the Bayesian structural time–series models to forecast the expected trends in annual alcohol‐related hospital admissions for 2020/21 (i.e. 5‐year forecasts), based on projected trends in population sizes and age distribution. Forecasts were created for geographical groupings that ranged from national level (broadest) to the Office for National Statistics (ONS) subgroup level. Area classifications and characteristics are described in detail elsewhere 15.

Briefly, the ONS area classifications cluster together the LTLAs that are the most similar, using a hierarchical method based on census statistics. Supergroups form the top tier of the hierarchy and provide the most generic description of the UK population. Groups form the middle tier and provide a more detailed summary of area characteristics, including a comparison of how they compare to the supergroup. Subgroups form the lowest tier, and offer the most detailed description of local characteristics.

### Data sources and description

Annual alcohol‐related hospital admission data for all 326 LTLAs were obtained from the Local Alcohol Profiles for England (LAPE) website for the midterm years 2003/04 to 2015/16 5. Several measures of alcohol‐related hospital admissions are available from LAPE, including ‘broad’ and ‘narrow’ measures. The broad measure counts all admissions for which at least one of the diagnoses is alcohol‐related. In contrast, the narrow definition counts only the admissions for which the primary diagnosis is an alcohol‐related condition, or a secondary diagnosis is an alcohol‐related external cause. Alcohol‐related admissions include those that are wholly or partially attributable to alcohol use. Hospital admissions are classified geographically by the patient's area of residence.

For our outcome, we used the ‘narrow’ definition of alcohol‐related hospital admissions, as it is less sensitive to variations in coding practices between hospitals and over time. Compared to the broad measure, however, the narrow measure may underestimate the overall burden of alcohol on health services 5.

Hospital admissions data were linked to observed mid‐year population data from the ONS for each LTLA 16 and also to projected figures for population size and age structure from 2014 to 2039 17. For modelling purposes, data were aggregated to eight strata: 0–15, 16–24, 25–34, 35–44, 45–54, 55–64, 65–74 and 75+ years of age.

### Statistical methodology

We implemented the Bayesian structural time–series models using the *bsts* 18 and *CausalImpact* 19 packages in R (version 3.2.4).

Bayesian structural time–series have been described in detail elsewhere 13, 20, 21. In brief, they are stochastic state‐space models that can incorporate trend, seasonality and regression components, spike‐and‐slab regression for optimal covariate selection and Bayesian model averaging to produce the final forecast. Two linked equations describe (a) how the outcome, in this example alcohol‐related hospital admissions, are related to an underlying latent state, and (b) how the latent state changes over time. Errors are normally distributed with a mean of zero and assumed to be independent. The regression component facilitates the construction of the modelled time–series, based on combinations of the covariates.

Our model was a local‐level model with regressors. We did not include a seasonal component, as our interest was in annual alcohol‐related hospital admissions. Because of the limited length of each time–series, we used a static framework in which the regression coefficients were fixed and did not include an additional linear trend component. A spike‐and‐slab prior was placed on the regression coefficients, where the ‘spike’ determines the probability that a covariate has a non‐zero coefficient based on independent Bernoulli distributions and the ‘slab’ is a weakly informative Gaussian prior with a large variance. The model averages over all combinations of predictors to construct an outcome time–series from the covariate time–series, which is weighted by Bayesian model averaging of the marginal inclusion probabilities for each regression coefficient.

The posterior predictive density is a joint distribution over all data points 13, and the posterior distribution of the model parameters was estimated using a sequence of Gibbs sampling steps from a Markov chain with a stationary distribution; a technique known as ‘stochastic search variable selection’ 21. Conditional on the latent state, which is sampled using Kalman filtering, the time–series and regression components are independent.

The posterior distribution of the time–series can then be projected forward, based on the known time–series data and the projected time–series, using the regression component of the model.

### Modelling strategy

Nowcasting here was defined as using the measured time–series from 2003/04 to 2012/13 to nowcast the outcome 1 year (to 2013/14), 2 years (to 2014/15) and 3 years (2015/16) ahead, and similarly using the measured 2003/4 to 2010/11 time–series to facilitate a 5‐year nowcast (to 2015/16). Differences between measured and nowcasted alcohol‐related hospital admissions were interpreted as nowcasting errors. Subsequently, we carried out forecasting using the same technique, but used the measured 2003/04–2015/16 time–series to forecast 5 years ahead to estimate the unmeasured 2020/21 alcohol‐related hospital admissions.

We linked the outcome data to the predictor data for 324 of 326 LTLAs. The City of London and Isles of Scilly were excluded due to missing data and small population sizes. The impact of the two excluded LTLAs on nationally aggregated alcohol‐related hospital admissions was minimal.

Prior distributions for the variance were set as Gamma distributions, and for the local linear trend models, the incremental error in the state was assumed a priori to be small and set to $\frac{1}{\sigma^{2}}\sim G\left(10^{-2},10^{-2}s_{y}^{2}\right)\;$, where $s_{y}^{2}=\sum_{t}\left(y_{t}-\bar{y}\right)/\left(n-1\right)$. The remaining priors were specified as follows: the sample size was set to the length of the measured, pre‐forecasted time–series; the degrees of freedom were set to the length of the measured time–series minus 1; μ (i.e. the estimate of the number of alcohol‐related hospital admissions) was set to a normal distribution with: a mean of the ‘known data’ alcohol‐related hospital admission time–series, the initial value set to the first value of the time–series, variance as an inverse Gamma distribution with 1% of standard deviation (SD) and initial value of 5% of the SD, and an upper limit of 150% of the SD of the measured time–series. Based on a preliminary non‐Bayesian run for 10% of LTLAs, the number of model parameters (i.e. β) expected to be non‐null was set to 5 and the expected explained variance was 70%.

Markov chain Monte Carlo (MCMC) chains were evaluated visually by trace and density plots. Mixing, autocorrelation and inappropriate starting values were acceptable if the Raftery–Lewis dependence factor (I) < 5; chain stability was evaluated based on Geweke diagnostics and Heidelberger–Welch tests; and Durbin–Watson tests were used to evaluate residual correlation in one‐step prediction (nowcasting) errors 22. To ensure that all evaluation criteria were met, estimation was based on 20 000 MCMC samples following a 10% burn‐in period.

### Data visualization

Results are presented in tables and in geospatial maps. For the latter, we exported modelling results into the mapping and analytics platform, ArcGIS 23, and linked this to local authority district boundaries for England (2015) 24 for all 324 LTLAs.

## Results

### Nowcasting

Table 1 provides a summary of the nowcasting results, including an assessment of nowcasting accuracy and precision over 1–5‐year periods. Average accuracy was good at both the national and LTLA level, while precision was also relatively good at the national level, but only lower at LTLA level.

**Table 1:** Summary of nowcasting results for measured and nowcasted alcohol‐related hospital admissions (lower‐tier local authorities = 324).

	Nowcasting period^a^			
	1 year (2013/2014)	2 years (2014/15)	3 years (2015/16)	5 years (2015/16)
National‐level annual alcohol‐related hospital admissions				
Total measured	326 940	326 930	336 330	336 330
Nowcasted^b^	325 978	327 083	327 968	329 074
95% credible interval	304 920–345 295	313 846–344 956	301 595–347 412	294 650–358 556
Error^c^	−0.29%	+0.05%	−2.49%	−2.16%
Annual alcohol‐related hospital admissions (lower‐tier local area level)				
Average measured	1009	1010	1038	1038
Range measured	200–6290	200–6490	220–6790	220–6790
Nowcasted	1006	1010	1012	1016
95% credible interval	296–3180	293–3207	283–3248	247–3351
Range nowcasted	206–6580	212–6596	216–6655	213–6661
Error (IQR)^c^	−0.53% (−6.08%, +4.68%)	−1.60% (−7.06%, +6.47%)	−3.82% (−11.74%, +5.78%)	−3.34% (−15.06%, +9.98%)

a One to 3‐year nowcasts were based on the pre‐nowcast (measured) time–series from 2003/4 to 2012/13. The 5‐year nowcast was based on the pre‐nowcast (measured) time–series from 2003/4 to 2010/11;

b nowcasted for 324 lower‐tier local authorities (LTLA) individually and then aggregated up to national level;

c median difference between LTLA measured and nowcasted alcohol‐related hospital admissions [with interquartile range (IQR)].

**Figure 1:**
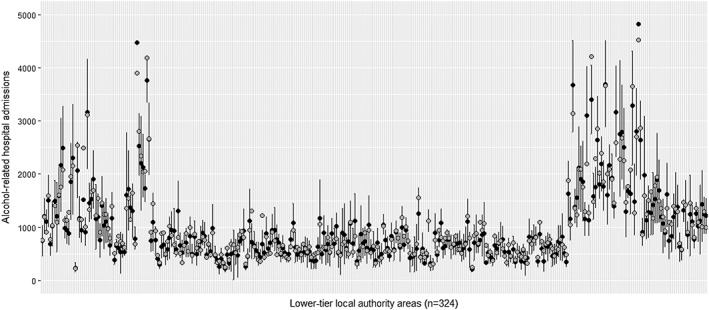
Comparison of local area measured (grey) and 5‐year nowcasted (black, with 95% credible intervals) alcohol‐related hospital admissions

For national estimates, average nowcasting errors ranged from +0.1 for the 2‐year nowcast to −2.5% for the 3‐year nowcast, with precision decreasing with increased nowcasting window; as expected. At the LTLA level, the average errors generally increased across the nowcasting period from −0.5% for the 1‐year nowcast to −3.8% for the 3‐year nowcast. For the 5‐year nowcast the average error was −3.3%. Similarly to the national‐level nowcasts, precision decreased with the increased nowcasting window. For the 3‐year nowcasts, errors for individual LTLAs ranged from −39.2 to +43.0% and were within 10% of the measured value for 174 of the 324 LTLAs. For the 5‐year nowcast errors ranged from −48.6 to +61.7% and were within 10% of the measured value for 132 of the 324 LTLAs. Figure 1 shows the measured and 5‐year nowcasted alcohol‐related hospital admissions and 95% forecasting credible intervals for each of the LTLAs for 2015/16, demonstrating that the accuracy and precision of nowcasting was generally good.

Least‐squares linear regression of 5‐year (2015/16) nowcasted and measured LTLA alcohol‐related hospital admissions resulted in a regression coefficient of 0.98 and *r* ^2^ of 98%.

### Forecasting

Table 2 shows the results of the 5‐year forecasts and population projections at the national level, based on the 324 LTLA‐level models. By 2020/21, national alcohol‐related hospital admissions were forecasted to increase by 3.3% nationally, to 334 411 (95% credible interval 313 846–344 956) annually. However, because the population is expected to increase by 3.7% during that same time–period, this corresponds to a reduction in the rate of alcohol‐related hospital admissions of 0.2 to 5.9% per 1000 people.

**Table 2:** Five‐year (2020/21) forecasted alcohol‐related hospital admissions and population size in England.

ONS population projections		
Population 2016 (measured, thousands)	55 219	
Population 2021 (projected, thousands)	57 248	
Change from 2016 to 2021	+3.7%	

Alcohol‐related hospital admissions	Number of admissions (95% credible interval)	Crude rate^a^ (LTLA range)
Total national alcohol‐related hospital admissions in 2015/16		
Measured (*n* = 324)	336 330	6.1 (3.3–11.5)
Nowcasted^b^	330 136	5.9 (3.4–11.4)
95% credible intervals	311 715–348 625	
Total national alcohol‐related hospital admissions in 2020/21		
Forecasted	334 411	5.9 (2.9–10.5)
95% credible intervals	313 846–344 956	
Forecasted average change relative to 2015/16 nowcasted values^c^	+3.3%	−0.2% (IQR −31.3%, +37.4%)

a Per 1000 people;

b note that this estimate differs from Table 1, because models used the 2003/4 to 2015/16 time–series rather than the 2003/4 to 2012/13 time–series that were used in the nowcasting phase;

c average of modelled lower‐tier local authorities (LTLA) changes; not weighted by population size of LTLA.

### Geographical variation

**Figure 2:**
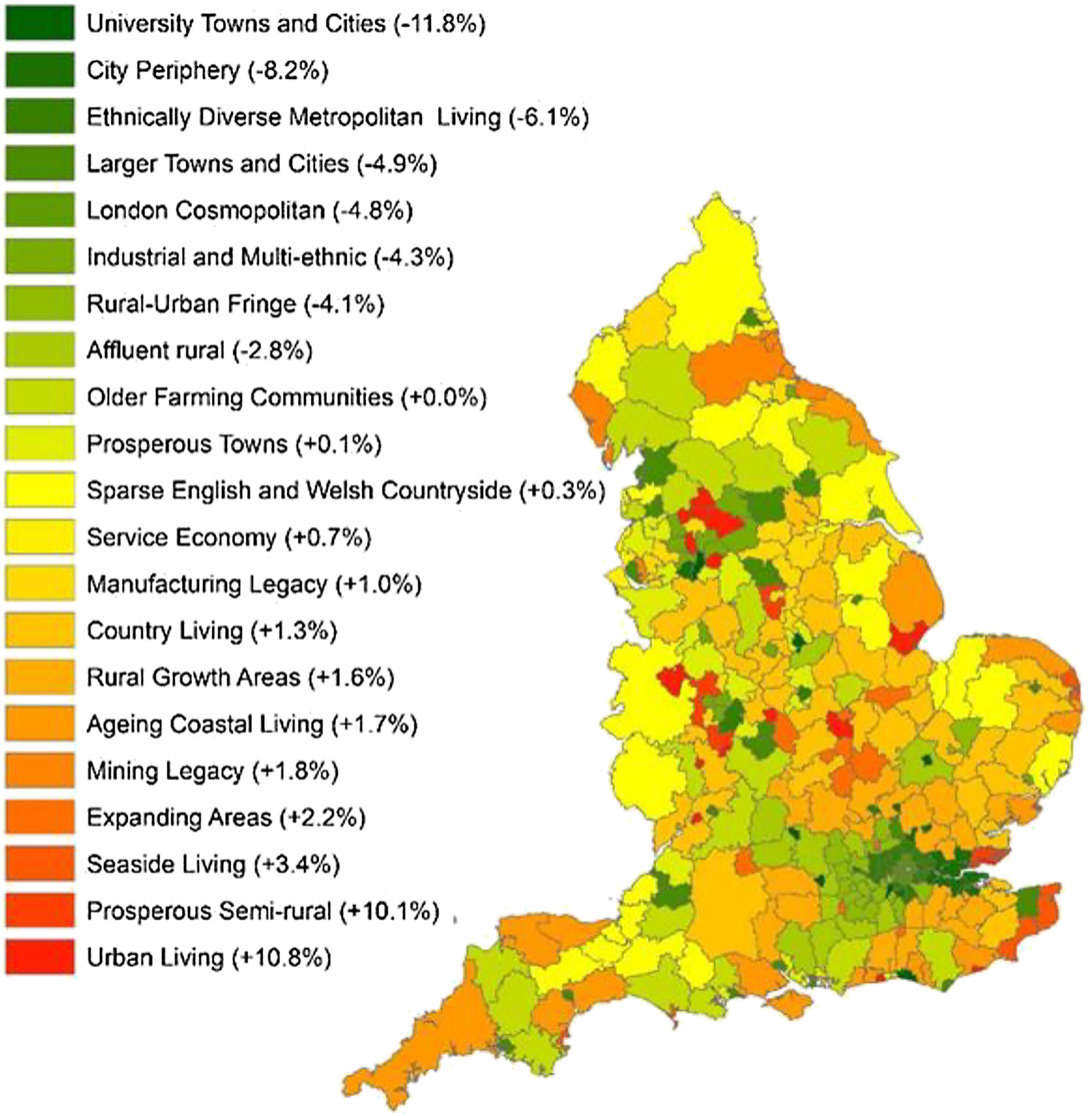
Forecasted change in crude rate of alcohol‐related hospital admissions for 2020/21 (compared to 2015/16) by geographical subgroup. [Colour figure can be viewed at wileyonlinelibrary.com]

Aggregation at the regional level showed relatively large differences between areas (see Table 3). The largest increases in alcohol‐related hospital admissions by 2020/21 were forecasted in the North West, with 7.0 admissions per 1000 people (+0.1 additional admissions per 1000 people on average compared to 2015/16) and the East Midlands, with 6.5 admissions per 1000 people (+0.04 additional admissions per 1000 people on average). The largest decreases were forecasted in London, with −0.2 fewer admissions per 1000 people. At the ONS subgroup level (Fig. 2), the largest reductions in the rates of alcohol‐related hospital admissions were forecasted in university towns and cities [median = −11.8%, interquartile range (IQR) = –17.9 to +0.9], city periphery areas (median = −8.2%, IQR = –11.0 to −5.1), and ethnically diverse metropolitan living areas (median = −6.1%, IQR = –14.1 to +0.4). The largest increases were forecasted in urban living areas (median = +10.8%, IQR = +2.7 to +17.2), prosperous semi‐rural (median = +10.1%, IQR = –1.8 to +13.0) and seaside living areas (median = +3.4%, IQR = –3.8 to +14.3). The medians and IQRs of all strata in are provided in Supporting information, Table S1.

**Table 3:** Forecasted alcohol‐related hospital admissions for 2020/21 by geographical region.

	Number	Rate per 1000	Rate change compared to 2015/16 per 1000
Region	Median (range)	Median (range)	Median (range)
East	689 (299–1436)	5.19 (3.28–8.10)	−0.09 (−1.30, +0.96)
East Midlands	595 (213–2428)	6.54 (4.71–9.14)	+0.04 (−1.03, +2.14)
London	1201 (609–1780)	4.12 (2.92–5.57)	−0.21 (−1.27, +1.12)
North East	1661 (770–3940)	8.15 (6.21–9.27)	−0.07 (−1.23, +0.56)
North West	1054 (290–3806)	6.97 (4.92–10.54)	+0.13 (−1.48, +1.70)
South East	614 (311–1620)	4.84 (3.20–7.83)	−0.01 (−1.00, +1.47)
South West	733 (234–4082)	6.04 (4.85–9.19)	−0.04 (−1.10, +1.55)
West Midlands	791 (297–6532)	6.32 (3.94–8.61)	+0.03 (−2.38, +1.04)
Yorkshire and the Humber	1307 (289–4570)	6.38 (4.79–8.34)	−0.04 (−0.91, +1.41)

**Figure 3:**
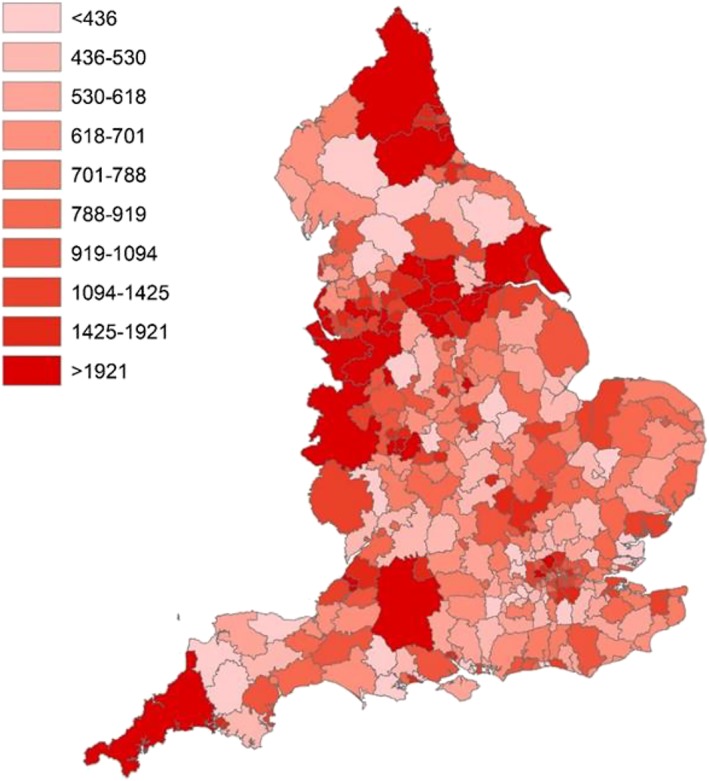
Forecasted annual number of alcohol‐related hospital admissions for 2020/21, for 324 lower‐tier local authorities (LTLAs). [Colour figure can be viewed at wileyonlinelibrary.com]

Figure 3 indicates the forecasted changes in the number of alcohol‐related hospital admissions for all 324 LTLA, demonstrating significant local variation. Supporting information, Figs S1–S3 provide further details of forecasted crude rates and changes at the LTLA level.

## Discussion

### Summary of results

Bayesian structural time–series models predicted national and LTLA level trends in hospital admissions well, on average, with errors ranging from +0.1% to −3.8% across nowcasting periods. However, there was greater variability in nowcasting accuracy between individual LTLAs. Nationally, the annual number of alcohol‐related hospital admissions was forecasted to increase by 3.3% (4275 admissions) by 2020/21, although the 95% credible interval included no change or even a decrease (95% CI = 313 846–344 956). Relative to the forecasted increase in the population during this period, this corresponded to a reduction in the crude rate of 0.2%. Regionally, the greatest increases in alcohol‐related hospital admissions were forecasted in the North West and East Midlands, with the greatest decreases in London and the East. At the population subgroup level, the largest increases in alcohol‐related hospital admissions were forecasted in urban living, prosperous semi‐rural and the service and industry legacy areas across the country. Increases were also forecasted in seaside and coastal areas.

### Interpretation of results

Results indicating higher expected increases in the more deprived urban and services, manufacturing and mining legacy areas are consistent with previous studies, which indicate that individuals of lower socio‐economic status and those who live in urban areas are disproportionally affected by alcohol‐related harm 25. Studies in England and Wales suggest that alcohol‐attributable mortality is up to five times higher among individuals living in the most deprived areas, compared to the least deprived 25, 26. Existing evidence indicates that these disparities cannot be explained by differences in alcohol consumption alone and, thus, this inequality is often referred to as the ‘alcohol harm paradox’ 27. Several explanations have been proposed to account for this apparent inequality, including the fact that individuals of lower socio‐economic status are more likely to experience concurrent exposure to multiple adverse behavioural risk factors (such as poorer nutrition, lower levels of exercise, greater levels of smoking and obesity), as well as barriers to accessing health‐care, which may exacerbate the adverse health effects of alcohol 27, 28. However, recent evidence suggests that alcohol‐attributable hospital admissions and mortality remain higher among individuals from disadvantaged areas, even after controlling for differences in alcohol consumption, smoking and body mass index 28. A further proposed explanation is reverse causation due to downward social selection, whereby individuals with hazardous alcohol use are more likely to move to deprived areas. However, a recent study found no evidence of selective migration to more disadvantaged areas among high‐risk drinkers 28. Overall, the precise mechanisms leading to socio‐economic inequalities in alcohol‐related harms remain poorly understood.

Our analyses also indicated greater forecasted increases in alcohol‐related hospital admissions in areas associated with inward migration of older individuals, including coastal and some rural areas. Data from 2016/17 demonstrate a clear age gradient in alcohol‐related hospital admissions increasing (per 100 000 population) from 301 for people aged under 40 to 887 for those aged 40–64 and 1013 for those aged 65 and over 29. Furthermore, studies from the Institute for Public Policy Research indicate that 90% of individuals aged 65 and over who make long‐distance moves from London relocate to coastal or rural areas 30. This pattern of migration, in conjunction with increased life‐expectancy, is likely to significantly contribute to future increases in health‐care burden in coastal and rural areas.

Reductions in alcohol‐related hospital admissions were forecasted in areas with a greater proportion of younger people and ethnic diversity, including university towns and cosmopolitan London. These trends are consistent with the continued migration of younger people to cities to seek employment and to access higher education, combined with the outward migration of individuals aged 40 and over 16, 30. They are also consistent with wider evidence of declining levels of alcohol consumption among younger people 31. Forecasted increases in more deprived areas, however, reflect the observation that some disadvantaged youth subgroups have not followed this trend of reduced consumption 31.

There has been a consistent disparity in the health of individuals in the north compared to the south of England, particularly for alcohol‐related harms 32. Our results indicate that in the absence of intervention, this trend is likely to continue and that disparities will increase further.

Finally, results were mixed for more affluent and rural areas. Forecasts suggested decreases in alcohol‐related hospital admissions in affluent rural and rural‐fringe areas and increases in prosperous towns and semi‐rural areas.

### Strengths and limitations

An important advantage of the Bayesian structural time–series methods that we applied, compared to traditional static methods of regression, is the use of Bayesian modelling averaging to make forecasts minimally dependent on specific hypothesized (and therefore potentially incorrect model specifications). This methodology automatically weights model factors based on their inclusion probabilities, thus selecting the most informative parameters, and does not require assumptions of linearity in the regression component of the model. The Bayesian framework allows for the incorporation of prior knowledge, which enables more accurate representation of the certainty of the estimates, and will evolve (improve its accuracy) over time. This work could also be extended to include a dynamic regression framework in which the regression coefficients are allowed to vary over time, subject to sufficient data 13, 33.

Our model predictions were based on the past behaviour of the time–series and demographic changes alone. This offered a parsimonious approach and we achieved good levels of accuracy on average, particularly for national estimates. However, nowcasting accuracy varied between LTLAs.

Bayesian structural time–series methods offer promising opportunities for further refinement, as they can accommodate a vast number of potential covariates and facilitate automatic selection of the most informative predictors. Further research that investigates the impact of adding additional factors to reduce errors in areas with lower nowcasting accuracy is warranted. In undertaking this research, we found that freely available and reliable forecasting data that would be informative in relation to alcohol‐related hospital admissions were limited. Nevertheless, some relevant time–series, such as alcohol taxation 34 and ethnic population 35 trends and forecasts, are available. We plan to investigate the impact of adding these data to our now‐ and forecasting models.

Comparison of the current methodology to naive autoregressive (AR) 1 modelling in the context of nowcasting consumer sentiment indicated a 14% improvement in one‐step‐ahead prediction errors when using Bayesian structural time–series 20.

A further specific benefit of this study is the validation of nowcasting models at LTLA level. As individual models were constructed for each LTLA, correlations between the predictors and outcomes were allowed to differ depending on local situations. Furthermore, this level of geography provides a foundation that enables aggregation to any level (including regional, supergroup, group and subgroup geographies). Thus, models can be tailored to support inferences about the probable effect of policies or public health trends for various population structures, as required. However, it is important to note that we implicitly assumed geospatial independence between LTLAs. Alcohol‐related hospital admission rates between adjacent LTLAs may not be independent (although there was little evidence of such in previous nation‐wide analyses 36, 37, and consideration of geospatial clustering may have improved the performance of our models.

An important limitation of this study is that the 2003/04–2015/16 time–series consists of only 13 measured data points on which to base forecast (down to only eight when 5‐year nowcasts for 2015/16 were produced). However, the nowcasting analyses in this paper show that despite this limited number of data points, the accuracy of 1–5‐year forecasts was reasonably good in most areas, even with only eight data points in the training data set, most probably as a result of Kalman filtering and Bayesian model averaging. Indicative comparable analyses using ARIMA models based on eight data points (similarly automated, using a random walk (0, 1, 0) model specification for all models and without Kalman filtering) showed much better accuracy for the Bayesian models than the ARIMA models, with root mean‐squared error of 224.5 and mean absolute error of 154.8 compared to 9165.1 and 2432.3, respectively (data not shown).

The association between demographic changes and alcohol‐related hospital admissions will be potentially confounded by multiple factors, which will differ between different communities. Although these confounders could be specifically modelled if the data were available, the local area‐specific modelling that we used allowed different associations to be modelled for each area, thereby attenuating the impact of local confounding factors.

Forecasts are a trade‐off between model freedom and statistical power. Therefore, priors for the standard deviations of variance were based on preliminary evaluations and the optimization of prediction errors. The precision of these estimates would also be important for, for example, informing policy decisions.

Now‐ and forecasting accuracy can also be affected by inaccuracies in the measured values of the predictors and outcome. In particular, it is important to note that the now‐ and forecasts here are principally based on population projections from the UK Office of National Statistics and, therefore, the quality of the forecasts relies on the accuracy of the population projections.

Finally, these forecasts do not provide information about the causal mechanisms that underlie the associations between hospital admissions, demographic changes and other covariates.

### Potential implications

The methodology that we have presented in this study could provide an automated application for local public health policymaking. We have demonstrated that Bayesian structural time–series models can support effective and timely planning for allocation of public health resources and the prioritization of interventions to reduce alcohol‐related harm. Specifically, our results could be used to inform alcohol policies in the North West, prosperous semi‐rural and urban living areas, where the greatest increase in alcohol‐related hospital admissions are forecast. Price‐based policies may be particularly effective at reducing the growing health inequalities in areas with greater levels of deprivation, such as the North of England and urban settings 27, 38. However, as the forecasting precision varies between LTLAs, for policy decisions at this level it would be worth refining the models further with local information to further increase accuracy and precision.

In addition to forecasting population trends, this methodology has also been used to evaluate the causal impact of alcohol licensing policies on health and crime 14, and can also be used to predict whether public health interventions are likely to have positive or negative impacts. For example, evidence suggests that ‘upstream’ public health interventions, such as minimum unit pricing, are more likely to reduce health inequalities than ‘downstream’ interventions, such as educational campaigns, which focus on individual factors 27. These proposed interventions could be specifically tested within a Bayesian framework using the nowcasting methodology described in this research.

## Declaration of interests

M.H. reports personal fees from Gillead, MSD, Abbvie, outside of the submitted work. The remaining authors have no declarations of competing interests.

## Supporting information


**Table S1** Medians and interquartile ranges (IQRs) of percentage change in rates in 2020/21 compared to 2015/16 of LTLA alcohol‐related hospital admissions stratified by ONS subgroup.
**Figure S1** Forecasted crude rates (per 1000 people) of alcohol‐related hospital admissions for the year 2020/21 at LTLA level.
**Figure S2** Forecasted relative change (%) in the number of alcohol‐related hospital admissions from 2015/16 to 2020/21 at LTLA level.
**Figure S3** Forecasted relative change in the crude rate (per 1000 people) of alcohol‐related hospital admissions from 2015/16 to 2020/21 at LTLA level.
