# Supplementary material for: Forecasting the 2021 local burden of population alcohol‐related harms using Bayesian structural time–series
Source: Addiction. 2019 Mar 6;114(6):994–1003. doi: 10.1111/add.14568 (PMC6563459; doi:10.1111/add.14568)
Supplement: Supplementary file 1 — Table S1 Medians and interquartile ranges (IQRs) of percentage change in rates in 2020/21 compared to 2015/16 of LTLA alcohol‐related hospital admissions stratified by ONS subgroup. Figure S1 Forecasted crude rates (per 1000 people) of alcohol‐related hospital admissions for the year 2020/21 at LTLA level. Figure S2 Forecasted relative change (%) in the number of alcohol‐related hospital admissions from 2015/16 to 2020/21 at LTLA level. Figure S3 Forecasted relative change in the crude rate (per 1000 people) of alcohol‐related hospital admissions from 2015/16 to 2020/21 at LTLA level. [file ADD-114-994-s001.pdf]

Online Supplementary Materials: Forecasting the 2021 local burden of population alcohol-related harms using Bayesian structural time-series

McQuire, C, Tilling, K, Hickman, M, de Vocht, F.*

*** Corresponding author:** Dr Frank de Vocht. Population Health Sciences, University of Bristol. Canynge Hall, 39 Whatley Road, Bristol. BS8 2PS.
Email: frank.devocht@bristol.ac.uk

Table S1. Medians and interquartile ranges (IQRs) of percentage change in rates in 2020/21 compared to 2015/16 of LTLA alcohol-related hospital admissions stratified by ONS subgroup

ONS Subgroup	Median change in rate (%)	IQR
Affluent rural	-2·8	-9·2 to +0·2
Ageing Coastal Living	+1·7	-4·7 to +9·8
City Periphery	-8·2	-11·0 to -5·1
Country Living	+1·3	-3·7 to +11·8
Ethnically Diverse Metropolitan Living	-6·1	-14·1 to +0·4
Expanding Areas	+2·2	-3·6 to +12·2
Industrial and Multi-ethnic	-4·3	-10·1 to +1·6
Larger Towns and Cities	-4·9	-9·3 to -1·7
London Cosmopolitan	-4·8	-12·4 to +0·8
Manufacturing Legacy	+1·0	-0·6 to +8·0
Mining Legacy	+1·8	-1·3 to +6·1
Older Farming Communities	+0·0	-5·9 to + 7·7
Prosperous Semi-rural	+10·1	-1·8 to +13·0
Prosperous Towns	+0·1	-2·5 to +8·0
Rural-Urban Fringe	-4·1	-10·2 to -1·1
Rural Growth Areas	+1·6	-5·8 to + 5·8
Seaside Living	+3·4	-3·9 to + 14·3
Service Economy	+0·7	-6·3 to + 3·6
Sparse English and Welsh Countryside	+0·3	-5·5 to + 3·1
University Towns and Cities	-11·8	-17·9 to + 0·9
Urban Living	+10·8	2·7 to +17·2

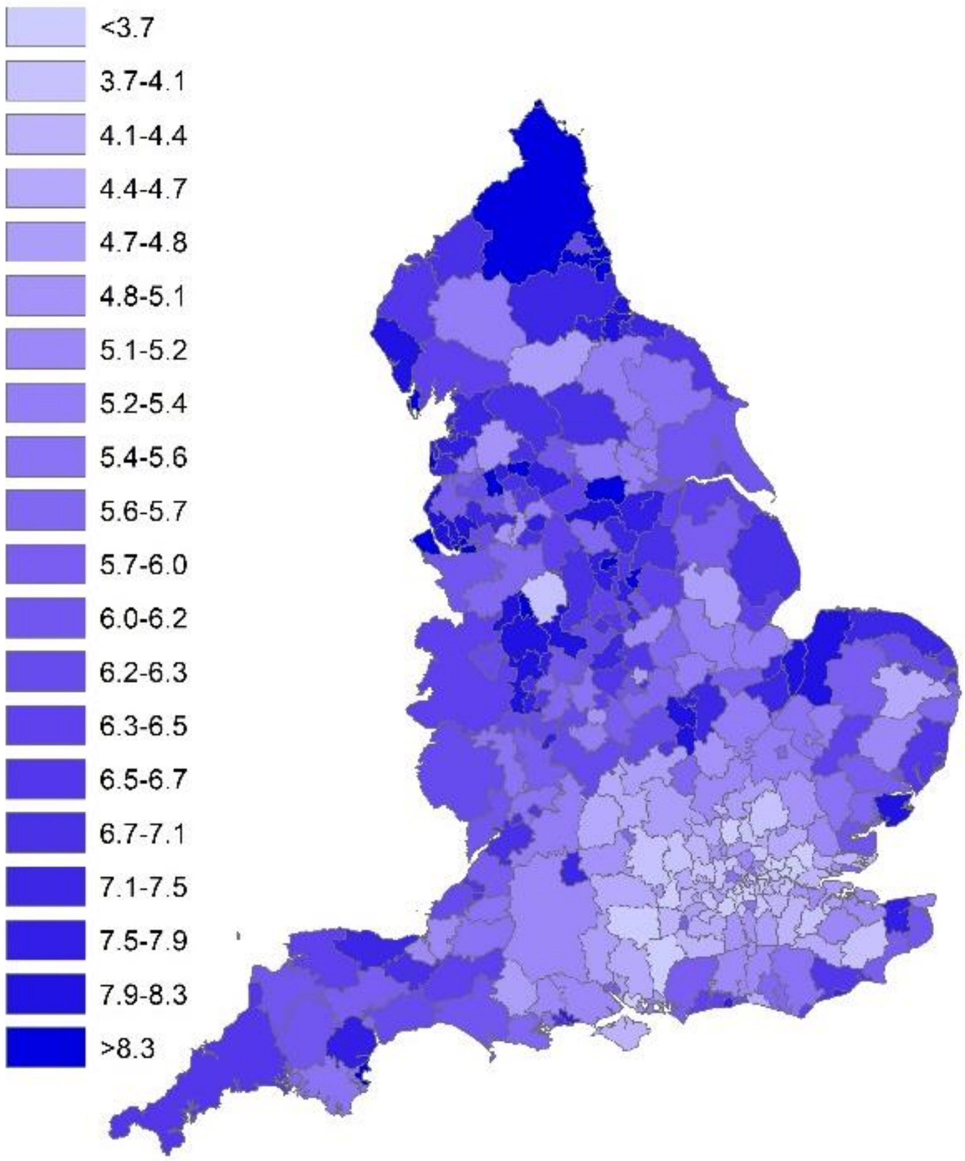

Figure S1. Forecasted crude rates (per 1,000 people) of alcohol-related hospital admissions for the year 2020/21 at LTLA level.

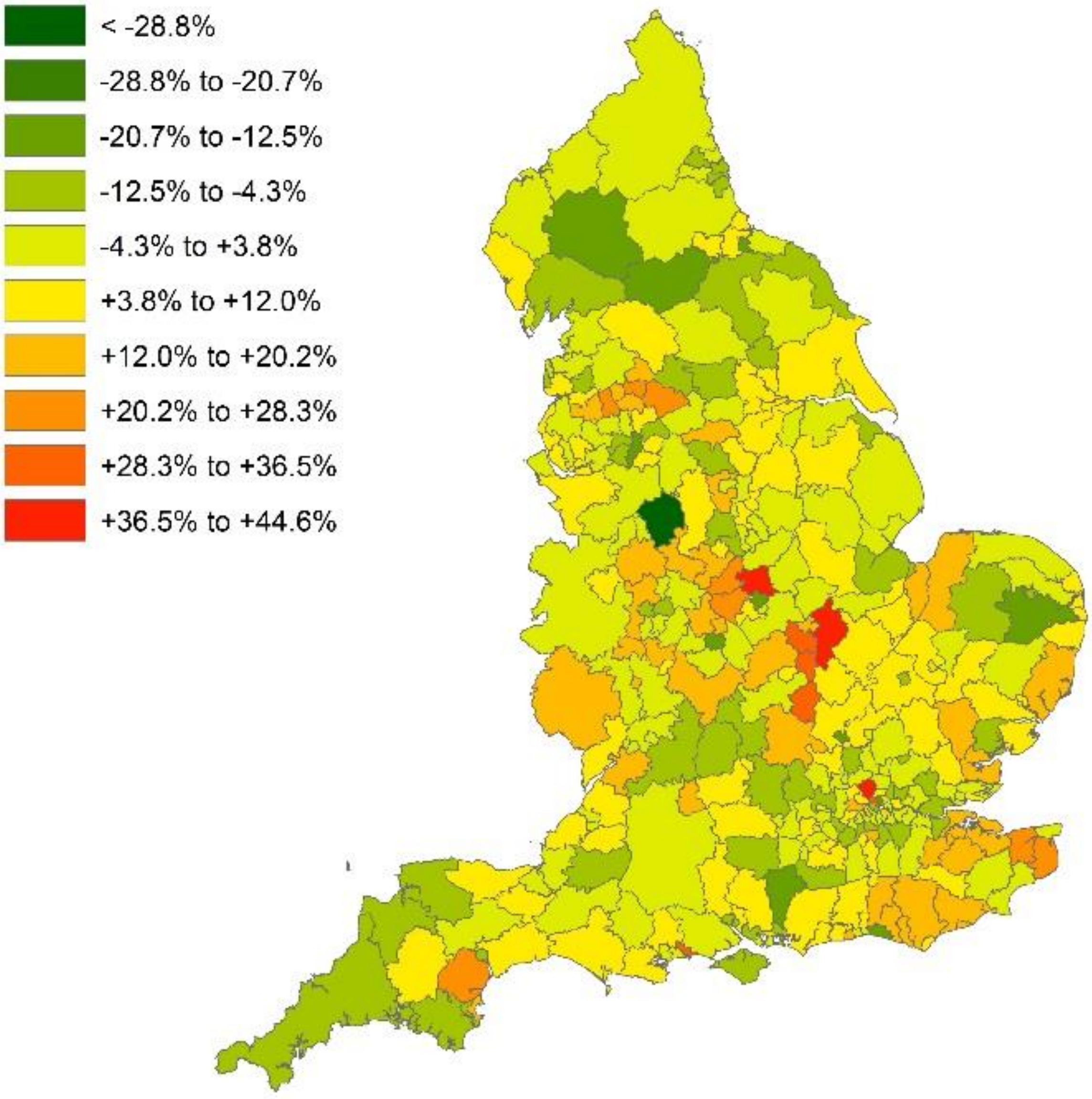

Figure S2. Forecasted relative change (%) in the number of alcohol-related hospital admissions from 2015/16 to 2020/21 at LTLA level.

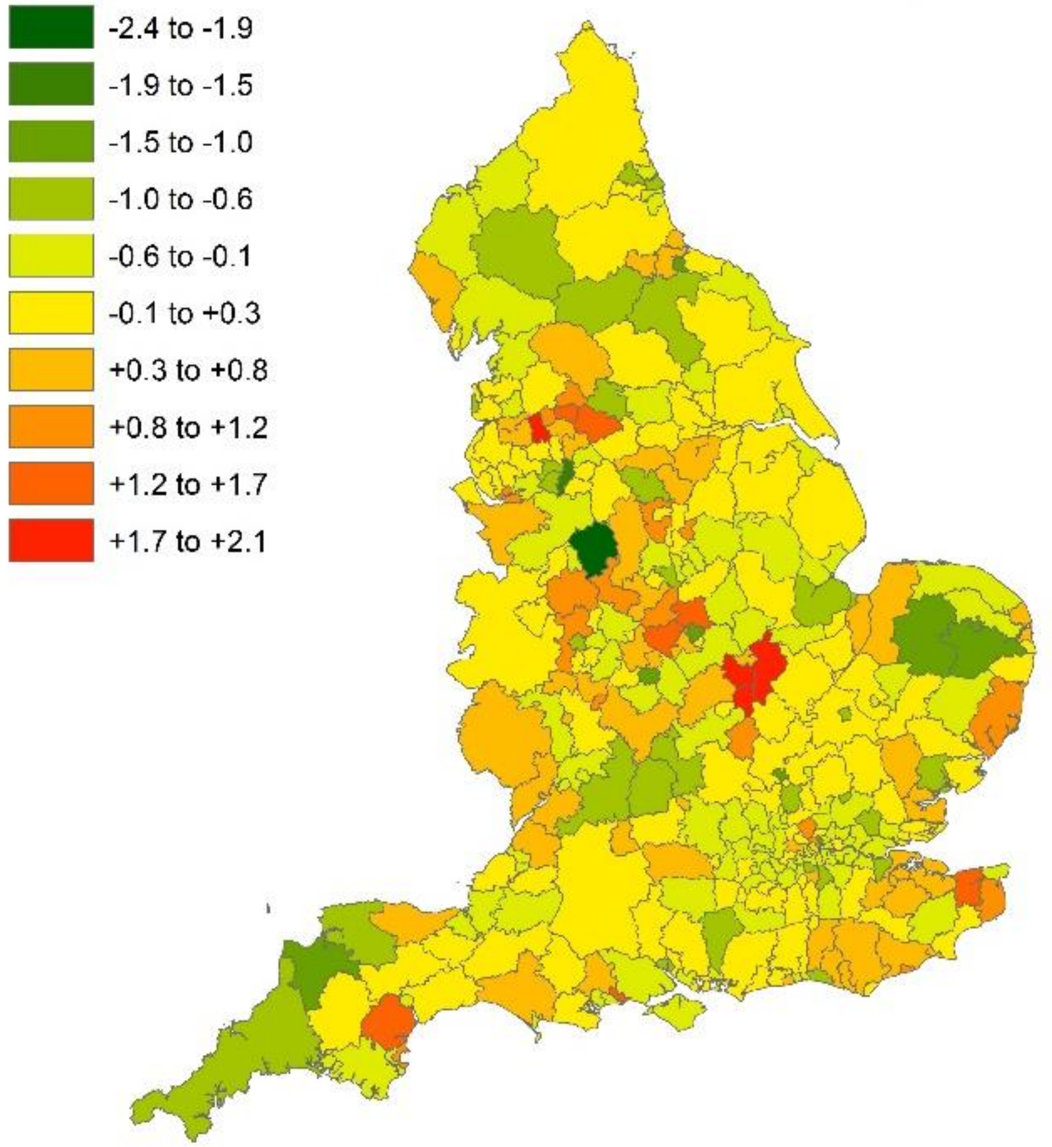

Figure S3. Forecasted relative change in the crude rate (per 1,000 people) of alcohol-related hospital admissions from 2015/16 to 2020/21 at LTLA level.
